# Model-Assisted Spleen Contouring for Assessing Splenomegaly in Myelofibrosis: A Fast and Reproducible Approach to Evaluate Progression and Treatment Response

**DOI:** 10.3390/jcm14020443

**Published:** 2025-01-12

**Authors:** Arman Sharbatdaran, Téa Cohen, Hreedi Dev, Usama Sattar, Vahid Bazojoo, Yin Wang, Zhongxiu Hu, Chenglin Zhu, Xinzi He, Dominick Romano, Joseph M. Scandura, Martin R. Prince

**Affiliations:** 1 Department of Radiology, Weill Cornell Medicine, New York, NY 10022, USA; ars4017@med.cornell.edu (A.S.); tjc4002@med.cornell.edu (T.C.); hrd2001@med.cornell.edu (H.D.); uss4002@med.cornell.edu (U.S.); yiw4017@med.cornell.edu (Y.W.); zsh4001@med.cornell.edu (Z.H.); chz4009@med.cornell.edu (C.Z.); xih4004@med.cornell.edu (X.H.);; 2 Department of Biomedical Engineering, Cornell University, Ithaca, NY 14853, USA; 3 Richard T. Silver Myeloproliferative Neoplasms Center, Weill Cornell Medicine, New York, NY 10065, USA; jms2003@med.cornell.edu; 4 Division of Hematology and Medical Oncology, Weill Cornell Medicine, New York, NY 10021, USA; 5 Department of Radiology, Columbia College of Physicians and Surgeons, New York, NY 10032, USA

**Keywords:** myelofibrosis, splenomegaly, deep learning, spleen volume reduction, clinical trials, treatment response, disease progression, reproducible, reliable, model-assisted contouring

## Abstract

**Background/Objectives**: Accurate and reproducible spleen volume measurements are essential for assessing treatment response and disease progression in myelofibrosis. This study evaluates techniques for measuring spleen volume on abdominal MRI. **Methods**: In 20 patients with bone marrow biopsy-proven myelofibrosis, 5 observers independently measured spleen volume on 3 abdominal MRI pulse sequences, 3D-spoiled gradient echo T1, axial single-shot fast spin echo (SSFSE) T2, and coronal SSFSE T2, using ellipsoidal approximation, manual contouring, and 3D nnU-Net model-assisted contouring comparing coefficients of variation. Changes in spleen volume were compared to all information to assess which measurement technique tracked disease progression with the greatest accuracy. **Results**: The coefficient of variation in spleen volume measurements averaging over 3 sequences was significantly lower for model-assisted contouring, 1.6% and manual contouring, 3.5%, compared to ellipsoidal estimation from 3 dimensions measured on axial and coronal T2 images, 15, *p* < 0.001. In 4 subjects with divergent treatment response predictions, model-assisted contouring was consistent with all information while ellipsoidal estimation was not. Manual contouring tracked similarly to model-assisted contouring but required more operator time. **Conclusions**: Model-assisted segmentations provide efficient and more reproducible spleen volume measurements compared to estimates of spleen volume from ellipsoidal approximations and improve objective determinations of clinical trial enrollment eligibility based upon spleen volume as well as assessments of treatment response.

## 1. Introduction

Myelofibrosis is a myeloproliferative disease characterized by progressive bone marrow fibrosis secondary to the hyperproliferation of abnormal myeloid cells [1]. These abnormal myeloid cells trigger bone marrow inflammation and fibrosis, increasing the workload of the spleen for extramedullary hematopoiesis and removing abnormal cells from circulation. The resulting splenomegaly is a hallmark feature that tracks disease activity. Increasing spleen volume not only indicates myeloid disease progression but independently contributes to deterioration in life quality causing abdominal pain, stomach compression with early satiety and weight loss, portal hypertension [2,3], sequestration of platelets with thrombocytopenia, and reduced physical activity [4].

Changes in spleen size signal disease progression or regression and spleen volume are used as a primary endpoint in myelofibrosis clinical trials [5,6,7,8]. The COMFORT-I and COMFORT-II trials, for example, established a 35% reduction in spleen volume (SVR35), measured using MRI or CT, as a clinically meaningful endpoint for assessing the efficacy of ruxolitinib in treating myelofibrosis [7,8]. In COMFORT-I, 41.9% of patients treated with ruxolitinib achieved SVR35 at 24 weeks compared to 1% in the placebo group (*p* < 0.001), while COMFORT-II reported 28% achieving this reduction at 48 weeks compared to none in the best available therapy group. Beyond symptom improvement, such as relief from abdominal discomfort and early satiety, pooled analyses of the COMFORT trials indicate that greater spleen volume reductions are associated with improved overall survival. Specifically, patients achieving SVR35 demonstrated reduced mortality compared to those with less than a 10% reduction in spleen volume [9]. Nevertheless, another study by Menghrajani et al. has questioned whether the survival benefit is directly causal and suggested that spleen volume reduction may primarily reflect patients with inherently better prognoses rather than a direct disease-modifying effect of the treatment. This, therefore, highlights the complexity of using spleen volume reduction as both a therapeutic endpoint and a surrogate marker of survival [10].

However, measuring spleen size by palpation can be unreliable since most of the spleen is under the rib cage; enlargement can only be inferred by palpating the inferior tip [11,12]. Several imaging methods are available to measure spleen size more directly. The simplest and most commonly used method is to measure the greatest dimension on coronal or axial CT or MRI images. A more accurate measure of spleen size can be estimated by measuring three orthogonal spleen dimensions and applying the formula for the volume of an ellipsoidal solid, providing an approximation for the spleen volume [13]. However, the spleen’s irregular shape, along with anatomical variations, makes it difficult to achieve exactly perpendicular measurements, increasing interobserver variability [12]. Direct spleen volume measurements can be obtained by tracing the spleen contour on each CT/MRI slice and summing the area multiplied by the slice spacing—a tedious, time-consuming, operator-dependent process. Recently, deep learning models have been proposed to automatically contour the spleen [2,14,15,16]. When an expert checks and corrects the model output, so-called “model-assisted contouring”, which may only take a few minutes, the benefit of manual contouring is achieved with a fraction of the time and effort.

The aim of this study was to compare the interobserver variability of spleen volume measurements in myelofibrosis patients for these four methods, ellipsoidal approximation, manual contouring, model-only, and model-assisted contouring, applied to abdominal MRI.

## 2. Materials and Methods

### 2.1. Patients

This Institutional Review Board-approved, HIPAA-compliant study retrospectively reviewed existing images and clinical data from 20 patients with biopsy-proven myelofibrosis. The requirement for informed consent was waived. Inclusion criteria included (1) bone marrow biopsy-confirmed myelofibrosis and (2) abdominal MRI performed within 2 months of bone marrow biopsy. Exclusion criteria were (1) unavailability of MRI with at least three sequences, including T2-weighted (axial and coronal) and axial 3D T1-weighted sequences, and (2) lack of clinical data within 3 months of the MRI.

### 2.2. Data Extraction

MRIs performed at 1.5T (*n* = 12) and 3T (*n* = 8) with imaging protocols listed in Supplementary Table S1 were extracted from the Picture Archival and Communication System (PACS). Patient clinical and laboratory data were retrieved from the electronic medical record (EMR) including demographic data, along with relevant laboratory findings and biopsy results corresponding to each imaging timepoint.

### 2.3. Deep Learning Model for Spleen Contouring

This study utilized a previously reported open-source deep learning-based 3D, multi-modality, multi-class model based on the nnU-Net framework trained with manually contoured spleen images, from 413 patients, subjected to rigorous quality control achieving a 0.97 Dice performance for spleen contouring [14] and available as an online calculator at www.traceorg.com. The model is an encoder–decoder network with skip connections to enhance detailed information in the deeper layers and gradient accumulation in the early layers. The encoder and decoder each contain seven levels. Each level is made up of two consecutive 3D convolutional layers, followed by an Instance Normalization layer and LeakyReLu activation. In most levels, 3D convolution layers down-sample feature maps by a factor of two.

### 2.4. Spleen Volume Measurements

**Figure 1:**
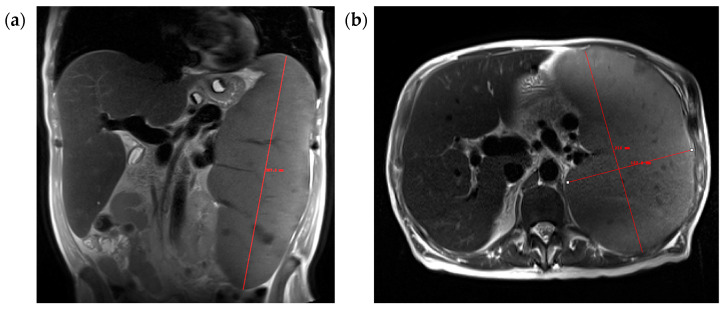
Spleen volume measurement using the ellipsoidal approximation from measurements of spleen length, width, and height: (**a**) height measured on coronal T2 and (**b**) length and width measured on axial T2.

Spleen volume measurements were performed independently by five expert observers (AS, VB, US, ZH, YW), using four different methods. These experts (1 radiologist, 1 nuclear medicine resident, 2 MDs in between medical school and residency, and 1 medical physicist) have been working on organ volume contouring for at least one year with experience contouring a minimum of 100 cases. First, the five experts independently, manually labeled the spleen on each image using ITK-SNAP software (version 3.8.0) [17], utilizing polygon and paintbrush tools. Second, spleen volumes were measured using a deep learning model without correction. Third, the same five observers manually corrected the spleen labels generated by the deep learning model, model-assisted contouring. Lastly, the observers measured the spleen’s width and length on axial T2-weighted images and the height on coronal T2 images, calculating spleen volume using the ellipsoidal solid formula [13] as shown in Figure 1:

Volume = Height × Length × Width × π/6

For each patient, spleen volume was calculated by averaging volumes obtained from axial and coronal acquisitions of T1- (axial only) and T2-weighted images. The manual and model-assisted methods were compared across identical pulse sequences by calculating the coefficient of variation across the five observers for the baseline scan of 20 myelofibrosis patients.

### 2.5. Comparison to Myelofibrosis Grade

For the first scan prior to any treatment, spleen volume was correlated with myelofibrosis grade on bone marrow biopsy for the temporally closest MRI measurement timepoint. Subjects who had treatments affecting spleen volume prior to bone marrow biopsy were excluded from this analysis. Correlation between spleen growth rate and myelofibrosis grade on bone marrow biopsy was also calculated where spleen growth rate was calculated as the slope on a spleen volume versus time curve.

### 2.6. Comparison to Dynamic International Prognostic Scoring System (DIPSS+)

The baseline, pretherapy scan was used to correlate spleen volume to the dynamic international prognostic scoring system (DIPSS+), based on a time-dependent risk evaluation calculated using the same day age, presence of constitutional symptoms, WBC count, hemoglobulin, presence of blasts in peripheral blood, karyotype, transfusion dependency, and platelets [18]. Next, similar steps were repeated for follow-up scans after the initiation of therapy including the calculation of the DIPSS+ score. Subjects with greater than the 1-year time interval between two consecutive scans or who did not have the necessary blood tests within 2 weeks of the MRI scan data were excluded from this analysis.

### 2.7. Clinical Trial Eligibility and Treatment Response Tracking

Myelofibrosis clinical drug trials typically require a minimum spleen volume of 450 mL to be eligible for enrollment [19]. Pre-therapy spleen volumes were compared across the four measurement techniques to assess their impact on clinical trial enrollment eligibility.

All patients enrolled in clinical trials (*n* = 14) with available follow-up scans at 3 months post-treatment initiation were evaluated to see how well each spleen volume measurement technique tracked each patient’s clinical course. Clinical and laboratory data at each time point were reviewed from the electronic medical record to assess treatment response. Changes in spleen volume relative to pre-treatment scans were measured using model-assisted and manual contouring, model-only, and ellipsoidal approximation, and these measurements were compared with corresponding clinical and laboratory data. These changes were classified as true positive, false positive, true negative, or false negative based on whether a ≥35% reduction in spleen volume occurred indicating a response. A spleen volume reduction of less than 35% was considered indicative of no response within the 3-month period following therapy initiation.

### 2.8. Statistical Analysis

Continuous variables were characterized as mean ± standard deviation for normally distributed data and as a median and interquartile range for non-normally distributed data. Normality was assessed using the Shapiro–Wilk test. The coefficient of variation was used to compare spleen volume measurements among the 5 observers for (a) manual contouring, (b) model-assisted contouring for axial T1, T2, and the average of all sequences, and (c) ellipsoidal volume estimates. The intraclass correlation coefficient (ICC) was calculated to assess interobserver agreement on spleen volume measurements across pulse sequences.

For the coefficient of variation between manual and model-assisted contouring, statistical significance was assessed using the F-Test Two-Sample for Variances (*p* < 0.05). Bivariate analysis was used to explore potential correlations between spleen volume and clinical parameters such as DIPSS+ score [18] and myelofibrosis grade (determined from bone marrow biopsy).

Sensitivity and specificity were calculated for the SVR35 prediction of response to therapy at 3 months post initiation of therapy.

## 3. Results

### 3.1. Study Population and Characteristics

Demographic data on 20 myelofibrosis patients diagnosed by bone marrow biopsy are demonstrated in Table 1. Of 20, 8 (40%) subjects were diagnosed with primary myelofibrosis. The other 12 of 20 (60%) were diagnosed with secondary myelofibrosis, the majority of which were secondary to polycythemia vera.

### 3.2. Coefficients of Variation and ICC: Ellipsoidal vs. Manual vs. Model Only vs. Model-Assisted

Median spleen volumes measured by the five observers for manual contouring, model-assisted contouring, model-only, and ellipsoidal approximation methods are shown in Supplementary Table S2. Spleen volume coefficient of variation when estimated from three linear measurements (length, width, and height) using the ellipsoidal approximation (Table 2) was 14.7%, significantly larger than the average from manually contouring three sequences, 3.2% (*p* < 0.001). Model-assisted contouring (averaging 3 sequences) had an even lower coefficient of variation, 1.6% (*p* < 0.001). The coefficient of variation for model-assisted contouring of individual sequences was even lower, 0.15% (axial T2), 0.22% (axial T1), and 0.20% (coronal T2), compared to manual contouring, 3.5%, 3.8%, and 3.1%, (*p* < 0.001 for all), respectively. This confirms that model-assisted contouring reduces variability across all three pulse sequences. The lower coefficient of variation for individual sequences undergoing model-assisted contouring compared to the three-sequence average likely reflects greater variation among T1 vs. T2 and axial vs. coronal image acquisition planes compared to the variation among five expert observers. The intraclass correlation coefficient (ICC) results, presented in Table 3, also demonstrate high agreement for both manual and model-assisted contouring methods across all pulse sequences but not as good for the ellipsoidal approximation.

### 3.3. Treatment Response Tracking

Within our cohort of 14 myelofibrosis patients in clinical trials with a regular 3-month follow-up, the change in spleen volume at 3 months after initiation of treatment showed varying sensitivity and specificity across the four different contouring methods. Ellipsoidal approximation achieved a sensitivity of 78% and a specificity of 60%. Manual contouring showed a sensitivity of 75% and a specificity of 83%. In contrast, both model-assisted contouring and model-only approaches achieved a sensitivity of 78% and a specificity of 100% for detecting treatment response (see Table 4).

### 3.4. Clinical Trial Eligibility

When the clinical trial eligibility was assessed, 14 of 15 (93%) patients were eligible for enrollment after measuring spleen volume using model-only and model-assisted contouring, and 15 of 15 (100%) were eligible using the ellipsoidal approximation (Table 4).

### 3.5. Effect of Spleen Volume Measurement Technique on Tracking Disease Activity

**Figure 2:**
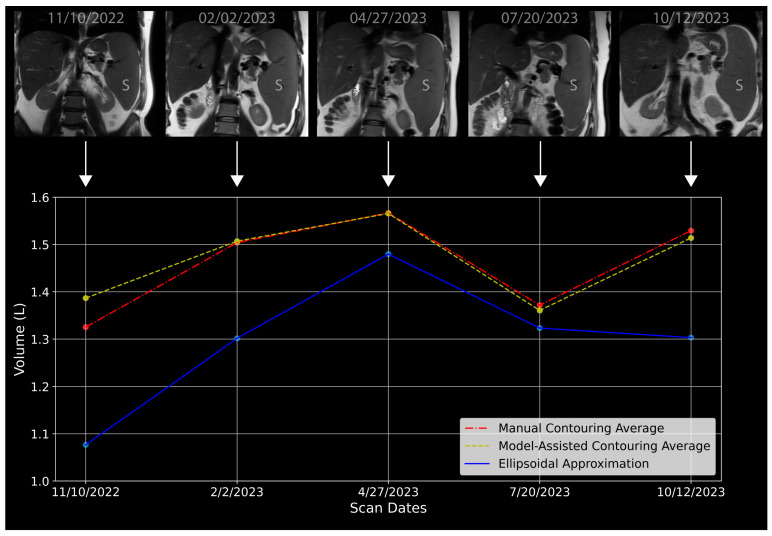
Spleen volume over time in a 63-year-old male with primary myelofibrosis, measured using three different methods: ellipsoidal approximation (blue line), averaging three sequences after manual contouring (red dash-dot line), and model-assisted contouring (yellow dashed line). Spleen (S) is depicted for each timepoint on the coronal T2 image at the top of the figure corresponding to 5 timepoints. The ellipsoidal approximation consistently underestimates spleen volume compared to contour-based methods. Notably, at the final timepoint, 10/12/23, the ellipsoidal approximation indicates a decrease in spleen volume, which contradicts the patient’s clinical deterioration, while the contouring methods show an increase in spleen volume, consistent with the worsening clinical status.

Over a 12-month period, a single observer (AS) measured spleen volume using manual, model-assisted contouring, and ellipsoidal approximation volume measurement methods, Figure 2. Both manual and model-assisted contouring for axial T1, axial T2, and coronal T2 sequences displayed similar trends, with spleen volumes increasing until month 3, decreasing by month 8, and then rising again by month 12. Model-assisted contouring showed less variability compared to manual contouring across all sequences. In contrast, the ellipsoidal approximation method consistently yielded lower spleen volumes compared to manual and model-assisted methods (Figure 2). For instance, in month 2, the ellipsoidal approximation method estimated a volume of 1.08 L, while manual and model-assisted contouring methods measured 1.38 L and 1.44 L, respectively. This pattern persisted throughout the 12 months, with the ellipsoidal approximation method showing more variability and consistently lower volumes, with a maximum of 1.30 L in month 3 compared to 1.56 L for manual and 1.51 L for model-assisted contouring. Furthermore, at month 8, the manual and model-assisted spleen volume trajectories showed an increasing spleen volume consistent with the patient’s worsening clinical course based upon all information, while the ellipsoidal approximation method showed a decreasing spleen volume in contradiction to the true disease activity.

### 3.6. Correlations Between Spleen Volume, DIPSS+ Score, and Myelofibrosis Grade

The correlation analysis between spleen volume and both DIPSS+ score and myelofibrosis (MF) grade (Supplementary Tables S3 and S4) did not reveal statistically significant associations for any of the measurement techniques. For example, Pearson correlation between spleen volume measured and DIPSS+ score yielded an R value of 0.12 (95% CI: −0.34 to 0.53, (*p* = 0.63)), indicating a weak, non-significant correlation. Similarly, the Spearman correlation between spleen volume and MF grade showed an R-value of 0.32 (95% CI: −0.16 to 0.68, (*p* = 0.17)), suggesting a positive trend that was not statistically significant. Additionally, the correlation between DIPSS+ score and MF grade resulted in a Spearman R-value of 0.27 (95% CI: −0.21 to 0.65, (*p* = 0.24)), further demonstrating the absence of a significant relationship between these parameters.

## 4. Discussion

Spleen volume for tracking myelofibrosis disease progression and response to treatment is routinely calculated from manual measurements of spleen length, width, and height on abdominal MRI using the formula for volume of an ellipsoidal solid. However, these data from 20 myelofibrosis patients demonstrate that estimating spleen volume using this ellipsoidal approximation is not as reproducible as manual or deep learning model-assisted contouring.

We found that when five expert observers measured spleen volume using three different approaches, splenic contouring reduced the coefficient of variation from 14.7% with the ellipsoidal approximation technique to 3–4% for manual contouring and less than 1% for a single sequence using model-assisted contouring. This reduction in the coefficient of variation affected determinations of clinical trial enrollment eligibility based upon spleen volume as well as determinations of treatment response based on 35% spleen volume reduction. Model-assisted contouring more accurately tracked the clinical trajectory based on all information without the tedious effort of manual contouring. These results are consistent with Azuri et al. who showed that the ellipsoidal technique had a mean error of 13.9 ± 9.6% for measuring splenomegaly on MRI in Gaucher disease compared to a mean error of 3.6 ± 2.7% for their deep learning approach trained on MRI scans from 30 patients [15]. Our superior deep learning performance likely reflects our use of an order of magnitude more MRI scans from over 400 subjects for model training.

Improved reproducibility of model-assisted contouring for organ volume measurements has also been demonstrated for kidneys and liver [14,20]. For example, in monitoring kidney volumes in ADPKD, the coefficient of variation for the ellipsoidal technique has been reported to be 15% to 17% [21,22] compared to 5.6% for manual contouring and 1.6% for model-assisted contouring. These numbers are similar to the coefficients of variation observed in the spleen, except for the manual contouring technique, which is lower in the spleen compared to polycystic kidneys (3.2% vs. 5.6%).

A reproducible method for measuring spleen volume in myelofibrosis is crucial, as changes in spleen volume serve as an important biomarker that influences clinical decisions. Accurate and timely information regarding therapy response, for example, when comparing spleen volume measurements using ellipsoidal approximation to model-assisted contouring, the divergence in spleen volume from the patient’s overall clinical picture further highlights the superiority of model-assisted contouring over ellipsoidal approximations. Automatic spleen labeling by deep learning models may have additional benefits beyond measuring volume. Realtime spleen labeling and tracking may assist hybrid MRI-guided LINAC for splenic radiotherapy in preparation before hematopoietic cell transplantation [23]. In these cases, the hybrid MRI-Linac systems with real-time spleen tracking and online adaptive treatment during irradiation may improve the targeting accuracy and minimize collateral damage to adjacent organs that may also be at risk.

The accuracy of volume measurements is a separate issue not investigated here because there is no absolute gold standard of reference available for spleen volume. However, Kodikara et al. [24] have demonstrated on phantoms that the ellipsoidal approximation method works best with more spherical and globular shapes and loses accuracy with elongated shapes typical of the enlarged spleens of myelofibrosis patients. Indeed, we observed that the ellipsoidal approximations systematically underestimated spleen volume compared to the contouring methods. Although we did not have an absolute gold standard of reference for assessing the accuracy of model-assisted contouring, there have been other reports in the literature demonstrating the superiority of organ contouring over ellipsoidal estimation. Wu et al. [25] reported that model-assisted contouring is more accurate than ellipsoidal estimation of prostate volumes compared to absolute measures of radical prostatectomy specimens in 310 patients.

We found that coronal sequences tended to yield more consistent measurements of enlarged spleens consistent with the recommendations of RECIL 2017 [26]. However, in some cases, axial sequences proved more reproducible, indicating that it is useful to analyze all MRI sequences available for each patient. The increased coefficient of variation with averaging multiple sequences for model-assisted contouring likely reflects increased variability between imaging sequences compared to the variability among five expert observers. Additionally, T2-weighted images generally produced larger volume estimates, while axial T1-weighted images exhibited a tendency toward smaller volumes. This variation reinforces the need for consistent methodology, when performing multiple sequential measurements. Measuring spleen volume on multiple MRI pulse sequences at each time point can be more reproducible by identifying and excluding or correcting outlier values and mitigating sequence bias by averaging [20]. Although 3T has a higher signal-to-noise ratio which can help with depicting organ boundaries, there are more artifacts at 3T including field inhomogeneity, coil issues, dielectric effects, and higher specific absorbed radiation (SAR). It was our impression that the 1.5T and 3T had similar performance.

CT has been proposed for measuring spleen volumes offering the advantage of higher through-plane (*z*-axis) resolution with greater volumetric coverage in a single breath hold. Deep learning models can also automate organ volume contouring on CT scans [27,28]. However, CT requires ionizing radiation limiting the frequency of measurements. With only a single acquisition, CT does not offer as robust of quality control as MRI providing multiple splenic volume measurements, one per MR imaging sequence. The unique splenic contrast on MRI compared to other organs makes identifying splenules more reliable compared to CT, which also improves spleen volume measurement accuracy by MRI relative to CT.

**Figure 3:**
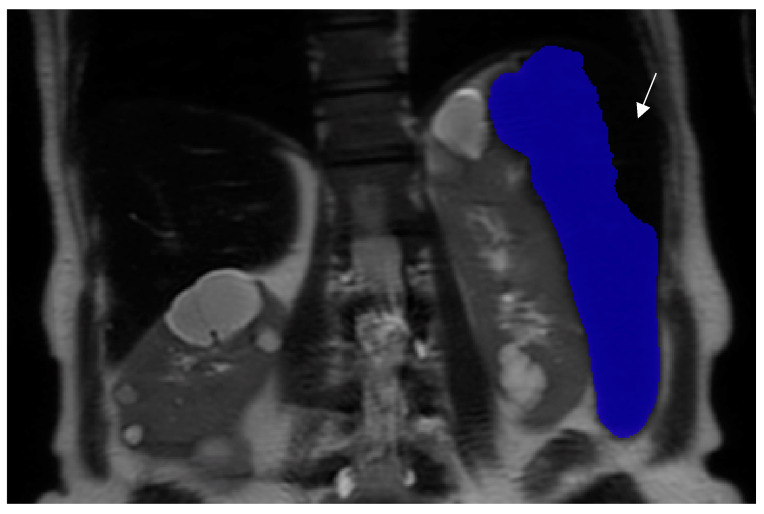
Suboptimal performance of the deep learning model in measuring enlarged spleen volume (blue segmentation) on a T2-weighted image, attributed to the dark spleen parenchyma (white arrow).

Although this study utilized expert observers, non-experts can use the model’s measurements without expert corrections. For spleen volume measurements that were performed automatically by the deep learning model without correction, the differences from expert corrections (model-assisted contouring) were minimal. Most cases showed a difference of less than 1%, with only a few exceptions where the difference exceeded 1%. Discrepancies in specific cases were likely attributable to the dark spleen parenchyma visible on T2-weighted images (Figure 3) occurring when high splenic iron levels spoil the MRI signal. These issues will likely be resolved once atypical cases like this are incorporated into the model training.

Harisinghani et al. [29] compared the prolate ellipsoid method with planimetry for spleen volume estimation in myelofibrosis, reporting a high correlation (R^2^ = 0.97) between the methods. However, compared to our data, several limitations emerge. The ellipsoidal method consistently underestimated spleen volumes in our study, especially in cases with anatomical complexities like splenules, a factor not addressed by Harisinghani et al. Additionally, Harisinghani et al. acknowledged the operator-dependent nature of the prolate ellipsoid method, which introduces variability. Our data, using a more precise manual and model-assisted contouring, demonstrated greater reproducibility. These differences highlight the limitations of the ellipsoid method in ensuring reproducible spleen volume assessments.

## 5. Conclusions

Percent spleen volume reduction is an important biomarker that has been accepted by the FDA for evaluating myelofibrosis disease progression and treatment response. Improving the accuracy and reproducibility of spleen volume measurements is critical for reliable clinical outcomes and therapeutic decision-making. Improving reproducibility decreases the time interval required to detect a meaningful change in spleen volume allowing for more frequent monitoring of patient response to treatment and earlier detection of treatment failures. Utilizing a model-assisted deep learning approach substantially enhances reproducibility, reducing observer variation in measuring splenomegaly in myelofibrosis patients.

## Figures and Tables

**Table 1:** Demographic data (at the time of MRI scanning) on 20 patients with myelofibrosis.

Parameter	Mean (±Standard Deviation) *
Patient (n)	20
Age **	69 (66–75)
Male–Female (%male)	12:8 (60%)
Race	
White	17 (85%)
Black	1 (5%)
Unknown	2 (10%)
Height (cm)	170 (±10)
Weight (kg)	69 (±14)
Genetic mutation ***	
JAK2	16 (80%)
CALR	4 (20%)
TET2	6 (30%)
ASXL1	5 (25%)
WBC	8.5 (4.9–14.3)
Platelet	
<100 × 109/L	4 (20%)
Blasts	
≥1%	20 (100%)
<1%	0
Transfusion dependent	
Yes	9 (45%)
No	11 (55%)
Spleen volume (mL)	988 (612–1317)

* Categorical variables are reported as numbers followed by percentages; continuous variables are reported as mean  ±  SD if normally distributed or median (interquartile range). ** Age at the time of the scan used to assess measurement reproducibility by 5 observers. *** Some patients had more than one mutation so the percentages total to >100%.

**Table 2:** Assessment of inter-observer variability by coefficient of variation for manual contouring, model-assisted contouring, and ellipsoidal approximation measurement methods that involved multiple observers. Note the model-only method was completely automatic and, therefore, had zero coefficient of variation because no observer was involved.

Coefficient of Variation	Ellipsoidal Approximation (%)	Manual Contouring (%)	Model-Assisted Contouring (%)	Model Only (%)
Axial T2	14.7 *	3.5	0.15	0
Coronal T2		3.1	0.20	0
Axial T1		3.8	0.22	0
Average of all sequences		3.2	1.6	0

* *p* < 0.001, compared to manual contouring and ellipsoidal approximation.

**Table 3:** Assessment of inter-observer variability by intraclass correlation coefficient for the manual contouring, model-assisted contouring, and ellipsoidal approximation methods each performed by five observers. Note the model-only method was completely automatic, and therefore, the calculation of the intraclass correlation coefficient is not applicable.

Intraclass Correlation Coefficient	Ellipsoidal Approximation	Manual Contouring	Model-Assisted Contouring
Axial T2	0.9665	0.9990	0.9999
Coronal T2		0.9990	1.0000
Axial T1		0.9973	0.9995
Average of all sequences		0.9987	0.9970

**Table 4:** Comparison of 4 spleen volume measurement techniques in assessing clinical trial eligibility (spleen volume > 450 cc) and response to therapy in 14 myelofibrosis patients using all information as the ground truth. For each spleen volume measurement technique, a reduction in spleen volume > 35% at 3 months follow-up was considered to be positive for response to treatment. Contouring and ellipsoidal approximation in selecting eligible participants for enrollment in clinical trials and assessing % change in spleen volume at 3 months post-therapy follow-up scans relative to clinical outcome. The total number of patients with post-therapy follow-up scans at 3 months was 14.

	Ellipsoidal Approximation	Manual Contouring	Model-Assisted Contouring	Model Only
* Number of patients eligible to enroll in clinical trial based on spleen volume > 450 mL	15	14	14	14
Response to Therapy				
True positive	7	6	7	7
True negative	3	5	5	5
False positive	2	1	0	0
False negative	2	2	2	2

* Total number of patients qualified to be enrolled in clinical trials with inclusion criteria requiring a spleen volume of >450 mL was 15.

## Data Availability

The data used for model training and validation are available and can be shared with a data sharing agreement on request from the corresponding author.

## Supplementary Materials

The following supporting information can be downloaded at https://www.mdpi.com/article/10.3390/jcm14020443/s1, Table S1: MR imaging parameters for the myelofibrosis cases at 1.5T and 3T using (A) GE Signa EXCITE and (B) Siemens MAGNETOM; Table S2: Descriptive stats (median and IQR) for the 20 subjects measuring spleen volume using manual contouring, model-assisted contouring, model-only, and ellipsoidal approximation. Reader 1: AS, Reader 2: US, Reader 3: VB, Reader 4: YW, and Reader 5: SH; Table S3: Dynamic International Prognostic Scoring System plus (DIPPS+) Scores among 20 patients with myelofibrosis at the time of scanning; Table S4: Myelofibrosis grade (from bone marrow biopsy) among 20 patients at the time of scanning.
