# Supplementary material for: Model-Assisted Spleen Contouring for Assessing Splenomegaly in Myelofibrosis: A Fast and Reproducible Approach to Evaluate Progression and Treatment Response
Source: J Clin Med. 2025 Jan 12;14(2):443. doi: 10.3390/jcm14020443 (PMC11766003; doi:10.3390/jcm14020443)
Supplement: Supplementary file 1 [file jcm-14-00443-s001.zip › jcm-3429341-supplementary.pdf]

Supplemental Table S1. MR imaging parameters for the myelofibrosis cases at 1.5T and 3T using (A) GE Signa EXCITE and (B) Siemens MAGNETOM.

**(A)** 1.5T (n=2) and 3T (n=4) (GE Signa EXCITE, Waukesha, WI)			
Parameter	**Coronal T2**	**Axial T2**	**Axial T1**
Sequence	SSFSE	SSFSE	LAVA
Fourier Space	2D	2D	3D
TR/TE/Flip	-/120/90	-/95/90	4/2/10
Matrix	(192 – 224) x 512	(192 – 224) x 512	192 x 512
Field of View (mm)			
x	440 – 480	320 – 450	300 – 460
y	440 – 480	320 – 450	300 – 460
Frequency (*x*) Resolution (mm)	0.6	0.6	0.6
Phase (*y*) Resolution (mm)	1.8 – 2.1	1.8 – 2.5	2.1
Slice Thickness (mm)	5	5	5
Spatial Resolution (mm^3)	5.6 – 6.5	5.6 – 7.8	6.5
Slice Spacing (mm)	6.0	6.0	2.5
**(B)** 1.5T (n=10) and 3T (n=4) (Siemens MAGNETOM, Erlangen, Germany)			
Sequence	HASTE	HASTE	VIBE DIXON
Fourier Space	2D	2D	3D
TR/TE/Flip	-/91/150	-/95/126	7/2/35
Matrix	(225 – 256) x (256 – 320)	(119 – 256) x (220 – 384)	(161 – 195) x (192 – 260)
Field of View (mm)			
x	375 – 480	341 – 416	351 – 442
y	381 – 480	283 – 416	298 – 403
Frequency (*x*) Resolution (mm)	1 – 1.3	1.6 – 3.4	1.2 – 1.7
Phase (*y*) Resolution (mm)	1.6 – 1.8	0.8 – 1.5	2.1 – 2.5
Slice Thickness (mm)	5 – 8	5 – 6	3 – 6
Spatial Resolution (mm^3)	1.6 – 10	1.1 – 10	1.3 – 4.0
Slice Spacing (mm)			

TR = Repetition Time. TE = Echo Time.

Supplemental Table S2. Descriptive stats (median and IQR) for the 20 subjects measuring spleen volume using manual contouring, model-assisted contouring, model-only and ellipsoidal approximation. Reader 1: AS, Reader 2: US, Reader 3: VB, Reader 4: YW, Reader 5: SH

**Height-adjusted Spleen Volume**		**Reader 1**	**Reader 2**	**Reader 3**	**Reader 4**	**Reader 5**	**All Readers**	**Model-only Volume**
Manual Contouring	Axial T2	968 (596 – 1366)	930 (581 – 1323)	1032 (641 – 1380)	968 (581 – 1350)	1014 (600 – 1344)	991 (603 – 1354)	836 (598 – 1227)
	Axial T1	983 (593 – 1290)	963 (601 – 1302)	1043 (637 – 1388)	957 (616 – 1323)	1001 (605 – 1236)	1110 (604 – 1327)	831 (567 – 1160)
	Coronal T2	948 (562 – 1293)	910 (551 – 1247)	987 (608 – 1295)	934 (555 – 1279)	951 (559 – 1272)	948 (561 – 1277)	830 (547 – 1256)
	Average of All Sequences	968 (958 – 975)	930 (920 – 947)	1032 (1009 – 1038)	957 (945 – 963)	1001 (976 – 1008)	968 (945 – 1008)	833 (553 – 1234)
Model Assisted Contouring	Axial T2	995 (622 – 1346)	1027 (622 – 1347)	1027 (622 – 1347)	1027 (621 – 1341)	1027 (622 – 1347)	1027 (1011 – 1027)	
	Axial T1	1013 (626 – 1295)	1015 (627 – 1320)	1016 (627 – 1320)	1016 (628 – 1337)	970 (627 – 1319)	1016 (1014 – 1021)	
	Coronal T2	924 (587 – 1277)	941 (589 – 1278)	943 (587 – 1278)	938 (585 – 1276)	938 (587 – 1277)	940 (928 – 943)	
	Average of All Sequences	995 (959 – 1004)	1015 (978 – 1021)	1016 (979 – 1021)	1016 (977 – 1022)	970 (954 – 998)	1015 (957 – 1021)	
Ellipsoidal Approximation	Axial & Coronal T2	978 (702 – 1348)	1026 (588 – 1220)	1235 (926 – 1585)	1026 (647 – 1258)	907 (737 – 1330)	972 (737 – 1330)	

Supplemental Table S3. Dynamic International Prognostic Scoring System plus (DIPPS+) Scores among 20 patients with myelofibrosis at the time of scanning.

DIPSS+ Score (1-6)	Estimated Median Survival Based on Score (Month)	Number of Patients (N)	Spleen Volume (mL)
0 – low risk	180	0	N/A
1 –intermediate-1 risk	80	1	532
2– intermediate-2 risk	35	5	1216
3- intermediate-2 risk	35	5	384
4-high risk		5	1122
5- high risk	16	2	2646
6- high risk		2	955

Supplemental Table S4. Myelofibrosis grade (from bone marrow biopsy) among 20 patients at the time of scanning

**Myelofibrosis Grade**	**Number of Patients**
Primary Myelofibrosis	
MF-1	2
MF-2	2
MF-3	3
Secondary Myelofibrosis	
Essential Thrombocytopenia	
MF-1	1
MF-2	1
MF-3	3
Polycythemia Vera	
MF-1	1
MF-2	4
MF-3	3
